# Caffeine-carbohydrate mouth-rinsing counter-acts an observed negative effect of mouth-rinsing procedure during sprint-endurance training performance in fasted athletes: A pilot study

**DOI:** 10.5114/biolsport.2022.109959

**Published:** 2021-11-10

**Authors:** Jad Adrian Washif, Kim Hébert-Losier, Karim Chamari, Christopher Martyn Beaven

**Affiliations:** 1 Sports Performance Division, National Sports Institute of Malaysia, Malaysia; 2 Te Huataki Waiora School of Health, University of Waikato, New Zealand; 3 Aspetar, Orthopaedic and Sports Medicine Hospital, FIFA Medical Centre of Excellence, Doha, Qatar

**Keywords:** Athletics, Ergogenic aid, Perceived activation, Ramadan, Sprint training

## Abstract

Carbohydrate mouth-rinsing has been reported to benefit endurance performance in athletes intermittently fasting; however, in the fasted state, the effects of combined caffeine and carbohydrate (CAF-CHO) mouth-rinsing on sprint-endurance performance are unknown. We determined the effects of CAF-CHO mouth-rinsing on kinetics, kinematics, and perceptual measures during a sprint-endurance performance commonly performed by track and field athletes in Muslim athletes fasting during Ramadan. In a randomised and counterbalanced single-blind study, ten national-level male sprinters and sprint/middle-distance runners (21.0 ± 2.0 y) participated in this study. They performed three sprint-endurance sessions on a non-motorised treadmill within the second and third weeks of Ramadan. Each session consisted of 3x15-s all-out sprints, with 2-min active recovery between each sprint. In each session, athletes either did not mouth rinse (NMR), or rinsed with 25 mL of CAF-CHO (4 g carbohydrate, 5 mg caffeine), or a placebo solution (PLA) prior to warm-up (30-min pre-trial), 1-min pre-trial, and mid-way through every recovery period. CAF-CHO maximised total sprint distance relative to NMR (210.3 ± 7.8 vs. 208.7 ± 9.1 m, d = 0.20), whilst counteracted the attenuation following PLA (204.6 ± 8.7 m; d = 0.66). Relative to NMR, CAF-CHO increased perceived activation prior to each sprint (p < 0.05, d = 1.23–2.05). Post-trial perceived exertion was lower for CAF-CHO (d = 0.12) and PLA (d = 0.58) compared to NMR (p > 0.05). Athletes indicated ‘no’ (50%) or ‘unsure’ (50%) whether mouth-rinsing would improve performance. The results suggest that CAF-CHO has a potential to optimise, and counter-act the negative effect of mouth-rinsing in Ramadan-fasted Muslims having a negative attitude towards this procedure.

## INTRODUCTION

Abstaining of fluid and food intake for extended hours can affect athletic performance [1]. This low-intake situation is consistently observed during the Ramadan intermittent fasting observed by healthy adult Muslims, in which the daily fast spans between dawn and sunset. The daily fast during the Ramadan month is variable, dependent on the latitude and seasonal variation, but commonly lasts 10 to 20 hours daily across ˜30 consecutive days. Varying levels of stress can be experienced during Ramadan, due to the alteration of biological clock, which affects the observant mentally and physically [2]. Elite soccer players are known to have strong negative beliefs and attitudes associating Ramadan fasting with poorer performance [3]. These psychological beliefs may impair exercise performance capacity [2, 4]. Several strategies have been advocated to cope with the training demands during Ramadan fasting, including managing training volume and intensity [2]; altering the training schedule to manage sleep loss [1]; adjusting to the night-time intake of food and fluid [5]; and mouth-rinsing [6, 7] to optimise athletic performance.

The effect of carbohydrate (CHO) mouth-rinsing on exercise performance has been increasingly studied [8]. Mouth-rinsing prior to and during exercise has been shown to improve endurance performance in running [9, 10] and cycling [6, 11], as well as repeated high-intensity bouts [12]. The primary mechanism of improved performance with mouth-rinsing has been attributed to activation of brain areas involved in reward stimuli as a result of oral receptors stimulation [11, 13], which improves motor drive or motivation [13, 14].

Some studies showing a positive effect of CHO mouth-rinsing involved exercise performance in a fasted state [14], which is not standard practice for athletes. Limited studies, however, are available concerning Ramadan fasting [6, 7], which numerous high-level athletes adhere to. Despite the rapid adaptation to fasting conditions, depletion of muscle and liver glycogen to maintain glucose levels can also occur quickly, and likely further deteriorate during exercise [15]. Che Muhamed et al. [6] examined the impact of CHO mouth-rinsing in a hot humid environment on participants observing Ramadan fasting and found improved endurance cycling performance, although the mechanism of performance improvement was not elucidated.

The beneficial effect of a combination of CHO and caffeine (CAF) mouth-rinses (CAF-CHO) on repeated high-intensity efforts has previously been demonstrated. Beaven et al. [12] reported that CHO enhanced peak power production, and that a CAF-CHO improved repeated sprint ability in a fed state. Caffeine is classified as a performance-enhancing stimulant [16], and its consumption results in lower perceived effort and enhanced contractile force, which subsequently improve athletic performance [17]. CAF mouth-rinse has additionally been shown to have beneficial effects on cycling endurance performance at moderate-intensity, muscle activity and fatigue tolerance among physically active men [18].

There are inconclusive findings seen in literature on exercise during Ramadan fasting, with some studies highlighted negative effects in high-intensity anaerobic performance [19]. To date, few studies have examined the effectiveness of mouth-rinsing during an anaerobic training regime. Sprint-endurance training involves effort bouts lasting 10 to 40 s at maximal speed, with 1 to 5-min rest periods. Such training pattern requires maintaining a high mechanical power and provides a potent stimulus for glycolytic activity necessary to evoke adaptive physiological changes important for sprint and middle-distance track athletes [20]. Thus, it is currently unknown whether mouth-rinsing with a combined CAF-CHO solution would benefit sprint-endurance training in athletes observing Ramadan fasting. Therefore, our primary purpose was to assess the effects of CAF-CHO mouth-rinsing on performance, kinetics, kinematics, and perceived exertion variables from a multiple sprint-endurance running session typically performed by sprinters. We hypothesised that mouth-rinsing and CAF-CHO solution would enhance sprint-endurance performance and perceptual measures compared to no-rinse and placebo conditions.

## MATERIALS AND METHODS

### Participants

Ten well-trained track and field athletes (six sprinters, four sprint/middle-distance runners) training at the National Training Centre in Malaysia completed the data collection during Ramadan in 2016. Athletes (mean ± standard deviation) were 21.0 ± 2.0 y, 1.71 ± 0.03 m, and 62.9 ± 4.8 kg with a competitive experience of 5.0 ± 1.6 y. The athletes had been training 6 to 9 sessions a week, 2 to 2.5 hours each session, and followed a periodised training programme to prepare for national and international events. Each athlete had experience of Ramadan fasting for over 10 years, which encompassed a daily fasting period of about 14 hours (abstaining from eating and drinking during daylight hours, ˜05:30 to ˜19:30). All athletes had been involved in various high-intensity sprinting for more than three years. An institutional research ethics committee provided approval for this study (RE/A/004/2018-24/2016), which was conducted in accordance with the Declaration of Helsinki. After understanding the risks and benefits of participation, all athletes gave their written informed consent to participate in this study.

### Study design

**FIG. 1:**
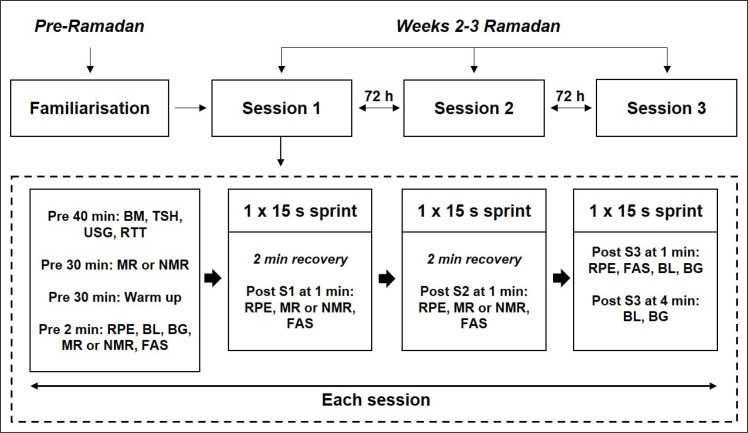
Schematic representation of data collection. BM, body mass; TSH, total sleep hour; USG, urine specific gravity; RTT, readiness to train; MR, mouth rinse; NMR, no mouth-rinse; RPE, rating of perceived exertion; BL, blood lactate; BG, blood glucose; FAS, felt arousal scale; S1/S2/S3, Sprint 1 or 2 or 3.

The present investigation involved a randomised, placebo-controlled counter-balanced crossover design, in which subjects made four visits to the laboratory (˜22 °C, ˜50% relative humidity): one familiarisation and three experimental. Figure 1 outlines the study protocol. The familiarisation session was carried out pre-Ramadan and involved a series of sub-maximal sprints on the treadmill similar to the experimental protocol. The subsequent three experimental sessions were carried out during weeks 2 and 3 of Ramadan, and separated by a minimum of 72 h. All sprint tests were conducted using the same non-motorised, instrumented treadmill sampling at 200 Hz (Woodway 3.0, Waukesha, Wisconsin, USA) [20]. For the experimental sessions, athletes reported to the laboratory at ˜16:00 for data collection, where the pre-experimental assessments, basic anthropometry, and experimental trials were performed. All athletes completed three experimental mouth-rinse conditions: a) CAF-CHO, b) a colour- and taste-matched placebo (PLA), or c) a no rinse control (NMR). All solutions were freshly prepared and kept in an insulated box containing ice during the test sessions. Throughout all trials, strong verbal encouragement was given for each subject. Athletes were also asked to guess what solution they had received after each test session.

The athletes were asked to maintain their regular training and to abstain from any intense exercise and caffeine intake in the 24 hours prior to each session. Athletes were also requested to standardise their meals during the 24 hours prior to each test, and instructed to replicate the first day’s diet at each experimental trial. During Ramadan, all athletes had similar lifestyles, as follows: a) time in bed was split into short sleeping periods, from about 00:30 to 04:30, 06:30 to 10:30, 14:00 to 15:30; b) first meal at sunset (iftar) (between 19:20 and 19:50), the second at midnight (23:00 to 23:30), and the third at dawn (05:00 to 05:30); c) one or two daily training sessions at 16:30 to 18:30 and/or 21:00 to 23:00. Only light to moderate training sessions were conducted before the first meal, with higher intensity training performed at night.

During each experimental session, the athletes performed a standardised warm-up (˜20-min including rest), which consisted of 3-min jogging on a motorised treadmill (at 8–10 km · h^−1^). Jogging was followed by 10-min of whole-body dynamic stretching (2 sets × 10 repetitions), 4-min of sprint drills (2 sets × 10-m of ankling, high-knee, back kick, and skipping), and 3-min of sub-maximal sprinting (3 × ˜5 s) on a non-motorised treadmill. Athletes then performed 3 × 15 s all-out sprints on a non-motorised treadmill, interspersed with 2-min active recovery between efforts (standing after the completion, and then walking while the perceptual measurements were taken).

### Biomechanical Measures

The distance, peak acceleration, peak velocity, peak horizontal force, and average velocity were recorded using the Force 3.0 software programme (Pacer Performance System, Joondalup, Australia). A laptop was interfaced with the four vertical load cells located under the running surface for vertical force measurement. A horizontal load cell connected to a non-elastic tether was attached via a vest allowing a slight forward lean position mimicking the sprinting posture. Load cells were calibrated using a range of known weights. Reliability data for speed and force measures have previously been reported with CV < 5% [21, 22].

### Physiological measurements

To measure metabolic responses, blood lactate concentration was measured using a portable lactate analyser (Lactate Scout, SensLab GmbH, Germany) at the following time points: immediately pre-, and 1-min and 4-min post exercise. Blood glucose concentration was determined from finger-prick samples taken at the same time points (Roche Diagnostics GmbH, Germany). Urine was collected prior to warm-up, and the urine specific gravity (USG) was analysed using a digital refractometer (Atago Co., Japan) to determine hydration status (Figure 1).

### Perceptual Measures

A series of perceptual measurements were recorded to assess the effects of mouth-rinsing on subjective experiences (Figure 1). The ratings of perceived exertion (RPE) were obtained using the 10-point scale. The RPE was recorded after warm-up, and 30-sec after Sprint 1, 2, and 3. A 6-point Felt Arousal Scale (FAS) was also administered to estimate the level of arousal/activation after mouth-rinsing. Ten minutes before the warm-up, athletes completed a pre-exercise assessment of readiness to train using a 100 mm visual analogue scale (VAS). Total sleep time and athlete’s ability to determine the colour and composition of the mouth-rinse data was collected via simple questionnaire. Additionally, participants were required to indicate “yes”, “no”, or “unsure” on a simple survey questionnaire to assess their perception on mouth-rinsing during Ramadan, whether it would improve performance. All participants agreed to perform mouth rinsing during Ramadan.

### Mouth-rinsing protocol

Athletes were asked to rinse with either CAF-CHO (solution containing 4 g carbohydrate and 5 mg caffeine), or a placebo [Tartrazine with CHO-free artificial sweetener, (PLA)] solution prior to warm-up (30-min pre-trial), 1-min pre-exercise, and in the middle (at 1-min) of each 2-min period of active recovery in-between sprints. The PLA content was formulated to ensure that it was colour- and taste-matched with the CAF-CHO solution and athletes were blinded to the composition. In accordance with previous research that has demonstrated positive effects of mouth-rinsing, 25 mL of solution was rinsed around the oral cavity for 10 s before expectoration [6, 8, 11]. For the NMR control trial, no fluid was provided.

### Statistical Analysis

Data are reported as mean ± SD unless otherwise stated. Data normality was checked using the Shapiro-Wilk test. One-way repeated measures analysis of variance (ANOVA) was used to detect significant differences between conditions for: (a) main dependent variables: distance, peak acceleration, peak velocity, peak horizontal force, and average velocity; (b) each of the pre-experimental conditions: USG and readiness to train. A series of two-way repeated measures ANOVA [condition (CAF-CHO vs. PLA vs. NMR) × time (e.g., pre vs. post)] was used to detect significant differences in the continuous variables or across multiple test series: sprint series of main dependent variables, blood lactate, blood glucose, RPE, and FAS. When sphericity was violated, Huynh-Feldt (ε > 0.75) or Greenhouse-Geisser (ε < 0.75) corrections were applied to adjust the degrees of freedom. When significance was reached, main effects for individual time points were further analysed using post-hoc analysis or paired t-tests, with Bonferroni adjustment for the number of pairwise comparisons. Statistical significance was set at p ≤ 0.05 for all analyses. All statistical analyses were performed using SPSS Version 16.0 (SPSS Inc., Chicago, IL, USA). The magnitude of mean differences was interpreted using Cohen’s *d* [23] effect size (ES), where ES of 0.20, 0.50, and 0.80 were interpreted as representing a *small*, *moderate*, and *large* change, respectively. An effect was deemed unclear if the 95% confidence interval overlapped the thresholds for both small positive and negative effects. Power analysis (G*Power 3.1, Franz Faul, Germany) showed that a sample size of nine would minimise the risk of incurring a type 2 statistical error, allowing detection of a *moderate effect* in five performance, kinetic, or kinematic measures being assessed with statistical power of 1-β = 0.80 and α = 0.05.

## RESULTS

### Pre-experimental condition

At the beginning of each trial, there were no significant differences (p > 0.05) in total sleep hours, training readiness, or hydration status (Table 1).

**TABLE 1:** Pre-exercise total sleep hour, readiness to train, and urine specific gravity

	NMR	PLA	CAF-CHO
Total sleep hours (h)	6.70 ± 0.67	7.15 ± 0.75	7.00 ± 1.83
Readiness to train (mm)	6.00 ± 1.70	5.60 ± 2.07	5.20 ± 1.69
Urine specific gravity (AU)	1.021 ± 0.005	1.020 ± 0.004	1.020 ± 0.007

NMR, no mouth-rinse; PLA, placebo rinse; and CAF-CHO, caffeine and carbohydrate rinse.

### Physiological measures

There was no interaction, and main effect of condition on blood lactate (p = 0.225 and 0.450, respectively) or blood glucose (p = 0.597 and 0.790, respectively); however, both increased linearly with *time* or number of sprints (p < 0.001) (Table 2).

**TABLE 2:** Physiological response before and after sprint-endurance trial during three different exercise conditions

	Pre-exercise	Post 1-min	Post 4-min
Blood lactate concentration (mmol · L^-1^)			
NMR	1.3 ± 0.5	12.4 ± 2.5*	13.8 ± 1.4*
PLA	1.2 ± 0.4	11.9 ± 2.7*	12.7 ± 1.5*
CAF-CHO	1.2 ± 0.5	12.5 ± 1.6*	13.9 ± 1.1*
Blood glucose concentration (mmol · L^-1^)			
NMR	4.7 ± 0.4	5.5 ± 0.7*	6.3 ± 0.6*
PLA	4.6 ± 0.3	5.2 ± 0.7*	6.5 ± 0.7*
CAF-CHO	4.6 ± 0.4	5.6 ± 0.7*	6.4 ± 0.8*

* Significantly different from baseline: p < 0.05; for both, no condition × time interaction.

NMR, no mouth-rinse; PLA, placebo rinse; CAF-CHO, caffeine and carbohydrate rinse.

### Sprint-endurance performance

Five athletes (50%) attained the highest total sprint distance during CAF-CHO, four (40%) during NMR, and one (10%) during PLA trials. There was no significant main effect of condition observed for total distance (p = 0.071). A higher total distance achieved in CAF-CHO (210.3 ± 7.8 m) compared to NMR (208.7 ± 9.1, *d*: 0.20, 0.8%) and PLA (204.6 ± 8.7, *d*: 0.66, 2.8%). The difference between NMR and PLA was *small* (*d:* 0.44, 1.9%).

**FIG. 2:**
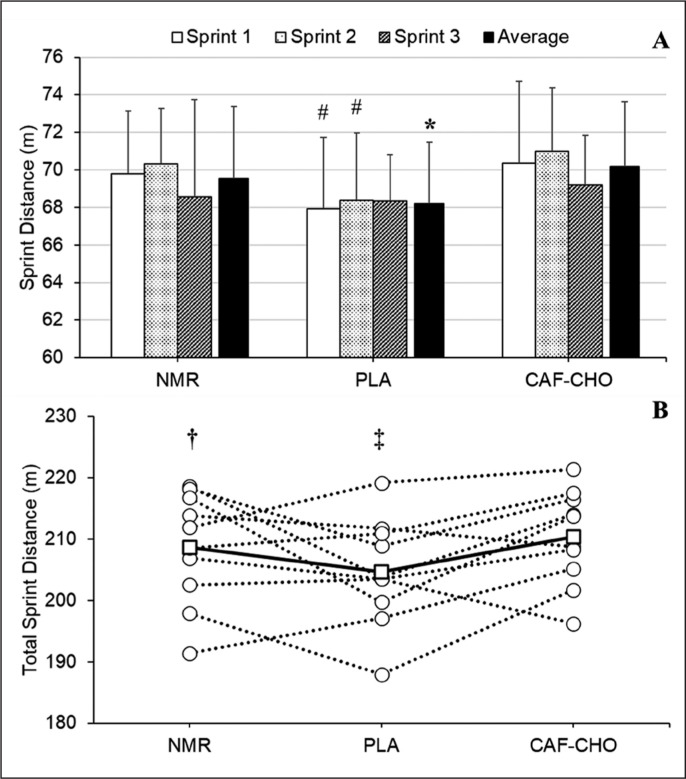
**A:** Sprint distances covered in Sprint 1, Sprint 2, and Sprint 3. **B:** Individual (white circles) and average (black circles) total distance covered during the 3 × 15 s sprint trials in the three conditions. NMR, no mouth-rinse; CAF-CHO, PLA, placebo rinse; caffeine and carbohydrate rinse; # *Moderate* difference from CAF-CHO and PLA; * *Moderate* difference from CAF-CHO; † *Small* difference from CAF-CHO; ‡ *Moderate* difference from CAF-CHO.

The performance in average distance was comprised of improved distances (*d*: > 0.50) in Sprint 1 and Sprint 2, but not Sprint 3 (Figure 2A). Individual distance data (total) are presented in Figure 2B.

Although comparison between the conditions did not reach significance for the other main dependent variables (peak acceleration, peak velocity, peak horizontal force, and average velocity), clear beneficial effects of CAF-CHO were observed for peak horizontal force in Sprint 2 relative to NMR (*d*: 0.78) and PLA (*d*: 0.70), and also average velocity in Sprint 1 (*d*: 0.59) and Sprint 2 (*d*: 0.74) vs PLA. Average velocity in the NMR condition during Sprint 1 was also clearly greater than the PLA condition (*d*: 0.64).

There were no significant effects observed regarding the condition by time (interaction effects) for average distance (p = 0.699) (Figure 2A), peak velocity (p = 0.248), peak acceleration (p = 0.970), peak horizontal force (p = 0.139), and average velocity (p = 0.512) (Table 3).

**TABLE 3:** Kinetics, and kinematics parameters of sprint-endurance performance

	Distance (m), ± SD	Peak Velocity (m/s), ± SD	Peak Acceleration (m/s^2^), ± SD	Peak Horizontal Force, (N) ± SD	Average Velocity (m/s), ± SD
NMR Average	69.6 ± 3.9	5.6 ± 0.4	5.8 ± 0.3	234 ± 24	4.6 ± 0.5
Sprint 1	69.8 ± 3.3	5.6 ± 0.3	5.7 ± 0.3	237 ± 22	4.7 ± 0.2
Sprint 2	70.3 ± 3.0	5.7 ± 0.3	5.8 ± 0.3	226 ± 19	4.6 ± 0.7
Sprint 3	68.5 ± 5.2	5.4 ± 0.4	5.8 ± 0.4	241 ± 29	4.6 ± 0.3

PLA Average	<M 00 + 1 <M 00 CO	5.5 ± 0.3	5.8 ± 0.5	243 ± 36	4.6 ± 0.2
Sprint 1	67.9 ± 3.8	5.5 ± 0.4	5.8 ± 0.3	256 ± 51	4.6 ± 0.3
Sprint 2	68.4 ± 3.6	5.4 ± 0.3	5.8 ± 0.5	232 ± 19	4.6 ± 0.2
Sprint 3	68.3 ± 2.5	5.4 ± 0.2	5.9 ± 0.7	242 ± 31	4.6 ± 0.2

CAF-CHO Average	70.2 ± 3.5	5.6 ± 0.3	6.0 ± 0.9	256 ± 67	4.7 ± 0.3
Sprint 1	70.2 ± 3.5	5.6 ± 0.3	6.0 ± 1.0	244 ± 46	4.7 ± 0.3
Sprint 2	71.0 ± 3.4	5.6 ± 0.3	6.1 ± 0.9	266 ± 86	4.8 ± 0.2
Sprint 3	69.2 ± 2.6	5.5 ± 0.2	5.9 ± 0.8	257 ± 70	4.6 ± 0.3

For all variables, no condition × time interaction was found. NMR, no mouth-rinse; PLA, placebo rinse; CAF-CHO, caffeine and carbohydrate rinse.

### Perceptual responses

**FIG. 3:**
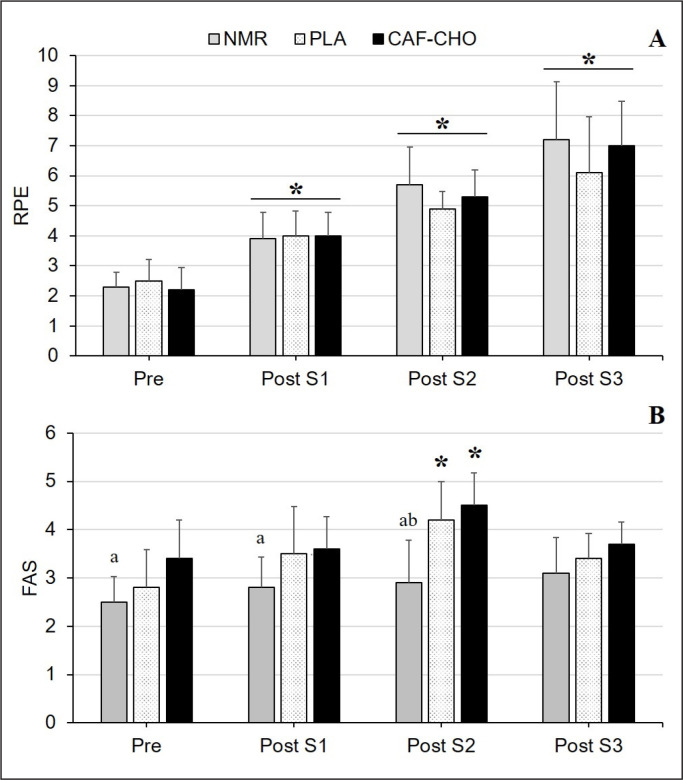
**A:** RPE (Rating of perceived exertion) and **B:** FAS (Felt arousal scale) throughout the performance test in the NMR, PLA, and CAF-CHO trials. NMR, no mouth-rinse; CAF-CHO, PLA, placebo rinse; caffeine and carbohydrate rinse. Note: Mouth-rinse was not administered for each condition after the last repetition (Post S3). * Significant difference in comparison to baseline (Pre), p < 0.05; ^a^ Significant between-group difference for CAF-CHO and NMR, p < 0.05; ^b^ Significant between-group difference for PLA and NMR, p < 0.05; Significant condition × time interaction for FAS, p < 0.05.

RPE increased linearly with time (p = 0.001), but there was no interaction (p = 0.060) and main effect of condition on RPE (p = 0.313) despite the observed values being lower by 3.0% (*d*: 0.12) for CAF-CHO and 15.3% (*d*: 0.58) for PLA compared to NMR (Figure 3A). A significant interaction (p = 0.001), condition (p = 0.001), and time (p = 0.001) effect was observed in the FAS, whereby the CAF-CHO condition exhibited higher perceived activation or arousal (p < 0.05, *d*: 1.23–2.05) than the NMR condition prior to each Sprint (Figure 3B). Post-trial RPE was ‘lower’ for CAF-CHO (*d*: 0.12) and PLA (*d*: 0.58) compared to NMR (p > 0.05). Furthermore, athletes indicated either “no” (50%) or “unsure” (50%) on mouth-rinsing perception during Ramadan.

### Solution identification

At the beginning of the experiment, all athletes were unsure whether any of the solutions or NMR would impact performance. Chi-squared analyses established that athletes were unable to distinguish the colour of solutions or correctly identify the CAF-CHO solution (p > 0.05).

## DISCUSSION

The primary finding of this study indicated that CAF-CHO resulted in a clear beneficial effect in sprint distances achieved in a typical sprint-endurance athletics training protocol when compared to PLA. Higher peak velocities obtained in the initial sprints drove the improved performance. Thus, the constituents of the CAF-CHO mouth-rinsing had a rapid (centrally-mediated) beneficial effect. However, the act of mouth-rinsing had a negative effect on performance as evidenced by the *small* favourable effect of NMR relative to the PLA. Overall, the positive effects of the CAF-CHO mouth-rinsing salvaged the negative effects of mouth-rinsing, but had a minimal and individual effects relative to the NMR condition.

The effects of mouth-rinsing in non-Ramadan conditions have been extensively studied, with improvement especially notable in endurance exercise lasting approximately 1 hour in fasted subjects [24]. There are also studies that have reported improvements in repeated sprint performance and power output [12], a 30-min running [9], moderate-intensity cycling to task failure [18], and a 2-km cycling time trial lasting ˜200 s that was incorporated into a protocol designed to mimic a competitive event [25]. However, a study of endurance-trained males reported no improvement in 1 hour cycling time trial during a postprandial or fed state [26]. Similarly, CHO mouth-rinsing does not improve multiple sprint performance and RPE during a field-based sprint test among physically active males [27]. Clearly, a number of variables could have influenced the potential ergogenic effects of mouth-rinsing, including fasting and training state of the participants.

It is important to note that the current study combined CAF and CHO solutions as a means of further developing the ergogenic effect of mouth-rinsing [12]. Beaven et al. [12] postulated that a contribution of central mechanisms to aspects of muscular function and sprint performance could be manipulated using a mouth-rinse intervention by combining CAF and CHO. Previous research has demonstrated the potential for rapid central effects of caffeine to impact on exercise performance [14]. Earlier investigations have similarly proposed central stimulation as the underlying mechanism of mouth-rinsing (i.e., CHO), which involves triggering of receptors located in the mouth [11, 13]. CAF mouth-rinsing activates the receptor cells (oropharyngeal epithelia) in the oral cavity, activating gustatory (i.e., taste) neural pathways to stimulate the same brain regions responsible for reward and information processing [28]. This effect would contribute to an increased motor drive [14] as a result of the enhanced central nervous system function, and in turn enhance muscle contraction, which is a prerequisite to improve exercise performance [13]. The results reported here are supported somewhat by earlier observations showing superior beneficial effects of a carbohydrate mouth-rinse on high-intensity running intervals when combined with caffeine ingestion than without [29].

A direct comparison with previous studies is limited due to paucity of research investigating sprint-endurance performance during Ramadan fasting. To date, CHO mouth-rinsing has yielded up to 12% improvement in cycling and endurance performances when compared to PLA or NMR conditions in Ramadan-fasting subjects [6, 10]. The performance improvement in biomechanical parameters in the present study are also consistent with the previous literatures that showed positive effects following a mouth-rinsing intervention [6, 7, 10, 11, 12, 18, 25]. A recent fasting study, however, found no statistically significant main effect of CHO mouth-rinsing on average power, average speed, and vertical stiffness during an interval sprinting protocol (2 sets of 5 × 5 s each, 25 s and 3 min recovery in-between sprints and sets, respectively), when comparing PLA and NMR [30]. This outcome contrasted studies that have found mouth-rinsing as a viable strategy to improve both cycling and running performances during the Ramadan fasting [6, 12]. The authors suggested that an insufficient volume and duration (10 mL rinsed for 5 s) of mouth-rinsing and also type of exercise performed for the lack of experimental effect [30].

CAF-CHO mouth-rinsing appeared to be beneficial in enhancing the kinematic parameters of sprint-endurance performance. Individual performance data suggested that the majority of participants attained best performances either during the CAF-CHO (50%) or NMR (40%) for the total distance, while only one (10%) of the participants attained best performance during the PLA (Figure 2B). Taken together, our findings indicate that sprint distance and velocities were enhanced by CAF-CHO during the sprint-endurance protocol; whereas, mouth-rinsing *per se* had a detrimental effect relative to a no-rinse condition. The lack of statistical difference between CAF-CHO and NMR in our study could have been due to the behavioural or society norms and beliefs regarding the practice of mouth-rinsing during the fasting ritual in Ramadan month. Different beliefs on mouth-rinse practice during Ramadan are an issue that needs clarification from religious scholars, as some permit the practice while others rule it as *Makruh,* or not recommended, during religious fasting [31]. Moreover, participants in the present study had a neutral or negative perception about the potential benefits of mouth-rising. Indeed, having negative attitudes and beliefs are considered constraints that could potentially affect performance [3] due to psychological consequence related to nocebo effect [2, 32]. This negative perception could have led to the observed decreased performance following PLA mouth-rinse; however, the constituents of CAF-CHO counteracted the observed negative effects of mouth-rinsing. It is also known that physiological response to caffeine administration is variable, with ‘caffeine responders’ identified in earlier studies that used CAF-CHO mouth-rinse [12]. Another potential explanation is the reported detrimental effect of the act of mouth-rinsing on exercise performance [33]. These authors reported a tendency for average power to be lower in the 10-s window of the mouth-rinse. It should be noted though, that in study of Gam et al. [33], rinsing was performed during exercise, as opposed to the rest periods in the current work. It is possible, however, that disruptions to the breathing patterns and other factors negatively affected the active rest obtained by our athletes. Importantly, the findings of the present study highlight the need for inclusion of NMR control condition when investigating mouth-rinsing effect.

The increment in perceptual responses over time appear to reflect the increased exercise demand across the three sprints (Figure 3), and an attenuation in the rise of RPE may well be interpreted as improvement in performance associated with mouth-rinsing protocols. However, this change must be carefully interpreted as reduced RPE may also indicate lower exercise effort. In the present study, the RPE recorded at the end of trials was higher in NMR when compared to CAF-CHO and PLA. An elevated RPE during NMR at the end of trials is comparable to a Ramadan study finding on cycling endurance [6] that also found significantly higher RPE in NMR compared to CHO mouth-rinsing. However, Dorling and Earnest [27] found that CHO mouth-rinsing did not influence RPE or other performance parameters, including the repeated sprint ability, compared to PLA rinsing. A subsequent study was also unable to detect between-group difference in RPE with CHO mouth-rinsing (vs PLA and NMR) in a repeated-sprint exercise in participants having fasted for 3 days (simulated Ramadan intermittent fasting) [30].

The present study also shows that arousal (as assessed by the FAS) was highest after Sprint 2 (or last mouth-rinse administration) during the CAF-CHO mouth-rinsing, which indicates a higher perceived activation when performing CAF-CHO compared to NMR condition. Additionally, a significant difference was also observed between CAF-CHO and NMR pre-exercise, indicating an elevated perceived arousal as a result of CAF-CHO mouth-rinsing prior to Sprint 1. Thus, the lower RPE and higher FAS during CAF-CHO were associated with an improvement in exercise tolerance and performance.

The present study was conducted 12–13 h into the fasting day, and at least 10 days after commencing the practice of Ramadan fasting. In such a situation, the participants’ endogenous glycogen would likely have been low [15]. However, no direct comparison was made between the participants’ non-Ramadan and Ramadan pre-exercise blood glucose concentrations. Such data would have provided additional evidence regarding the impact of Ramadan fasting. Nevertheless, the level of blood glucose concentration pre-trial (4.6 to 4.7 mmol · L^-1^) was similar in all conditions, and approximately 30% less than that reported by Che Muhamed et al. [6] in fasting athletes. Elevated glucose post-exercise has been previously attributed to a centrally mediated effect [25]. Importantly, the blood glucose concentration at the completion of each condition did not differ significantly between conditions, indicating that the observed performance differences were not mediated by blood glucose levels.

We acknowledge that this study has several potential limitations. Participants in this study were training with different coaches, and variation in the training volume between participants may have affected the individual sessions. Nevertheless, at least 24 h of rest was taken by each participant before the experimental sessions to eliminate the potential influence of fatigue on the study results, and training readiness and hydration status were similar across all conditions. Although we did not measure the expectorated fluid, the experiment was conducted in a strict methodological design, and the investigators were present to ensure adherence of protocol. Moreover, the expectorated fluid may have included a small volume of saliva, although it is unlikely swallowing small quantities of solution would have statistical influence [30]. We also note that the experimental group consisted of athletes taking part in a range of events (100 to 800 m), and that a more homogenous group may also have lowered the variability in responses and increased the likelihood of detecting differences between the conditions. We note that small differences have the potential to be meaningful in high-level athletes and the cumulative effect of improved training has the potential to influence adaptive outcomes. Further studies may replicate the experiments to establish the outcomes. Additionally, future studies may investigate the effects of mouth-rinsing for different fasting durations (e.g., 13 vs 18 hours into fasting), utilise a more homogenous sample, and include pre-Ramadan control condition, while considering female athletes.

## CONCLUSIONS

The present study indicated that CAF-CHO mouth-rinsing may be a viable strategy to counter-act the potential negative effects of mouth-rinsing on sprint-endurance performance observed in some Muslims who do not believe that mouth rinsing procedures are beneficial to performance. CAF-CHO did induce higher arousal (i.e., FAS) prior to the last repetition of sprint-endurance performance, and lower RPE which indicates perceptual benefits. Nevertheless, such an intervention should only be implemented on an individual-basis and accordance with accepted cultural practices.

### Practical Applications

The outcomes of the present study highlighted the potential benefit of CAF-CHO mouth-rinsing during Ramadan fasting for counter-acting the negative effect of mouth rinsing in athletes for whom mouth rinsing was detrimental. Therefore, mouth-rinsing is a viable option, and CAF-CHO mouth-rinsing can be effective as an ergogenic aid in challenging metabolic conditions (i.e., Ramadan fasting) for athletes training to optimise sprint-endurance performance. However, the implementation of such an intervention needs to be considered alongside individualised efficacy, the noted NMR results, and acceptance in cultural practices. Specifically, Muslim athletes and practitioners should consult religious scholars prior to undertaking mouth-rinsing given the potential to inadvertently swallow the rinsed liquid. Coaches are encouraged to pre-test athletes before Ramadan to identify the effectiveness of any mouth rinse intervention at an individual level.

## References

[1] Chaouachi A, Leiper JB, Chtourou H, Aziz AR, Chamari K. The effects of Ramadan intermittent fasting on athletic performance: recommendations for the maintenance of physical fitness. J Sports Sci. 2012; 30:53–73. 22738880 10.1080/02640414.2012.698297

[2] Chamari K, Rouissi M, Bragazzi N, Chaouachi A, Aziz A. Optimizing training and competition during the month of Ramadan: Recommendations for a holistic and personalized approach for the fasting athletes. Tunis Med. 2019; 97(10):1095–1103. 31691937

[3] Farooq A, Herrera CP, Zerguini Y, Almudahka F, Chamari K. Knowledge, beliefs and attitudes of Muslim footballers towards Ramadan fasting during the London 2012 Olympics: a cross-sectional study. BMJ Open. 2016; 6:e012848. 10.1136/bmjopen-2016-012848 PMC5051417 27670523

[4] Aziz AR, Che Muhamad AM, Roslan SR, Ghulam Mohamed N, Singh R, Chia MYH. Poorer intermittent sprints performance in Ramadan-fasted Muslim footballers despite controlling for preexercise dietary intake, sleep and training load. Sports. 2017; 5:4. 10.3390/sports5010004 PMC5969002 29910364

[5] Shephard RJ. Sport participation and Ramadan observance: Advice for the athlete. J Fast Health. 2015; 3(2):65–73.

[6] Che Muhamed AM, Mohamed NG, Ismail N, Aziz AR, Singh R. Mouth rinsing improves cycling endurance performance during Ramadan fasting in a hot humid environment. Appl Physiol Nutr Metab. 2014; 39:458–464. 24669987 10.1139/apnm-2013-0276

[7] Pak IE, Cuğ M, Volpe SL, Beaven CM. The effect of carbohydrate and caffeine mouth rinsing on kicking performance in competitive Taekwondo athletes during Ramadan. J Sports Sci. 2020; 2:1–6. 10.1080/02640414.2020.1735033 32122273

[8] de Silva TDA, de Souza MEDCA, de Amorim JF, Stathis CG, Carol Góis Leandro CG, Lima-Silva AE. Can carbohydrate mouth rinse improve performance during exercise? A systematic review. Nutrients. 2014; 6(1):1–10. 10.3390/nu6010001 PMC3916844 24451304

[9] Rollo I, Williams C, Gant N, Nute M. The influence of carbohydrate mouth rinse on self-selected speeds during a 30-min treadmill run. Int J Sport Nutr Exerc Metab. 2008; 18:585–600. 19164829 10.1123/ijsnem.18.6.585

[10] Bataineh MF, AL-Nawaiseh AM, Abu Altaieb MH, Bellar DM, Hindawi OS, Judge LW. Impact of carbohydrate mouth rinsing on time to exhaustion during Ramadan: A randomized controlled trial in Jordanian men. Eur J Sport Sci. 2018; 18(3):357–366. doi: 10.1080/17461391.2017.1420236. 29364063

[11] Carter JM, Jeukendrup AE, Jones DA. The effect of carbohydrate mouth rinse on 1-h cycle time trial performance. Med Sci Sports Exerc. 2004; 36:2107–2111. 15570147 10.1249/01.mss.0000147585.65709.6f

[12] Beaven CM, Maulder P, Pooley A, Kilduff L, Cook C. Effects of caffeine and carbohydrate mouth rinses on repeated sprint performance. Appl Physiol Nutr Metab. 2013; 38(6):633–637. 23724880 10.1139/apnm-2012-0333

[13] Chambers ES, Bridge MW, Jones DA. Carbohydrate sensing in the human mouth: Effects on exercise performance and brain activity. J Physiol. 2009; 587:1779–1794. 19237430 10.1113/jphysiol.2008.164285 PMC2683964

[14] de Ataide-Silva T, Ghiarone T, Bertuzzi R, Stathis CG, Leandro CG, Lima-Silva AE. CHO mouth rinse ameliorates neuromuscular response with lower endogenous CHO stores. Med Sci Sports Exerc. 2016; 48(9):1810–1820. doi: 10.1249/MSS.0000000000000973. 27128664

[15] Stannard SR. Ramadan and its effect on fuel selection during exercise and following exercise training. Asian J Sports Med. 2011; 2(3):127–133. 22375231 10.5812/asjsm.34760 PMC3289214

[16] Jenkinson D, Harbert A. Supplements and Sports. Am Fam Physician. 2008; 78(9):1039–1046. 19007050

[17] Tarnopolsky MA. Effect of caffeine on the neuromuscular system-potential as an ergogenic aid. Appl Physiol Nutr Metab. 2008; 33(6):1284–1289. 19088790 10.1139/H08-121

[18] Melo AA, Bastos-Silva VJ, Moura FA, Bini RR, Lima-Silva, AE, de Araujo GG. Caffeine mouth rinse enhances performance, fatigue tolerance and reduces muscle activity during moderate-intensity cycling. Biol Sport. 2021; 38(4):517–523. 34937960 10.5114/biolsport.2021.100147 PMC8670801

[19] Boukhris O, Hsouna H, Chtourou L, Abdesalem R, BenSalem S, Tahri N, Trabelsi K, Stannard SR, Chtourou H. Effect of Ramadan fasting on feelings, dietary intake, rating of perceived exertion and repeated high intensity short-term maximal performance. Chronobiol Int. 2019; 36(1):1–10. 30207750 10.1080/07420528.2018.1513943

[20] Iaia FM, Bangsbo J. Speed endurance training is a powerful stimulus for physiological adaptations and performance improvements of athletes. Scand J Med Sci Sports. 2010; 20 Suppl 2:11–23. 20840558 10.1111/j.1600-0838.2010.01193.x

[21] Brughelli M, Cronin J, Mendiguchia J, Kinsella D, Nosaka K. Contralateral leg deficits in kinetic and kinematic variables during running in Australian rules football players with previous hamstring injuries. J Strength Cond Res. 2010; 24(9):2539–2544. 19996776 10.1519/JSC.0b013e3181b603ef

[22] Hughes MG, Doherty M, Tong RJ, Reilly T, Cable NT. Reliability of repeated sprint exercise in non-motorised treadmill ergometry. Int J Sports Med. 2006; 27(11):900–904. 16739088 10.1055/s-2006-923791

[23] Cohen J. Statistical Power Analysis for the Behavioural Sciences. 2nd ed. Hillsdale (NJ): Lawrence Erlbaum Associates; 1988.

[24] Jeukendrup AE. Oral carbohydrate rinse: placebo or beneficial? Curr Sports Med Rep. 2013; 12(4):222–227. doi: 10.1249/JSR.0b013e31829a6caa. 23851408

[25] Luden ND, Saunders MJ, D’Lugos AC, Pataky MW, Baur DA, Vining CB, Schroer AB. Carbohydrate mouth rinsing enhances high intensity time trial performance following prolonged cycling. Nutrients. 2016; 8(9):E576. doi: 10.3390/nu8090576. 27657117 PMC5037560

[26] Beelen M, Berghuis J, Bonaparte B, Ballak S, Jeukendrup AE, van Loon LJC. Carbohydrate mouth rinsing in the fed state does not enhance time trial performance. Int J Sport Nutr Exerc Metab. 2009; 19:400–409. 19827464 10.1123/ijsnem.19.4.400

[27] Dorling JL, Earnest CP. Effect of carbohydrate mouth rinsing on multiple sprint performance. J Int Soc Sports Nutr. 2013; 10(1):41. doi: 10.1186/1550-2783-10-41. 24066731 PMC3849766

[28] Wickham KA, Spriet LL. Administration of caffeine in alternate forms. Sports Med 2018; 48:79–91. 29368182 10.1007/s40279-017-0848-2 PMC5790855

[29] Devenney S, Mangan S, Shortall M, Collins K. Effects of carbohydrate mouth rinse and caffeine on high-intensity interval running in a fed state. Appl Physiol Nutr Metab. 2018; 43(5):517–521. 29262272 10.1139/apnm-2017-0458

[30] Cherif A, Meeusen R, Ryu J, Taylor L, Farooq A, Kammoun K, Fenneni MA, Aziz AR, Roelands B, Chamari K. Repeated-sprints exercise in daylight fasting: carbohydrate mouth rinsing does not affect sprint and reaction time tests performance. Biol Sport. 2018; 35(3):237–244. 30449941 10.5114/biolsport.2018.77824 PMC6224849

[31] Che Muhamed AM, Singh R, Aziz AR. Carbohydrate mouth rinsing a novel approach to maintain exercise performance during ramadan fasting? J Nutr Fast Health. 2014; 2(4):162–164.

[32] Aziz AR, Che Muhamad AM, Roslan SR, Ghulam Mohamed N, Singh R, Chia MYH. Poorer intermittent sprints performance in Ramadan-fasted Muslim footballers despite controlling for pre-exercise dietary intake, sleep and training load. Sports. 2017; 5(1):4. 10.3390/sports5010004 PMC5969002 29910364

[33] Gam S, Guelfi KJ, Fournier PA. Opposition of carbohydrate in a mouth-rinse solution to the detrimental effect of mouth rinsing during cycling time trials. Int J Sport Nutr Exerc Metab. 2013; 23(1):48–56. 22952137 10.1123/ijsnem.23.1.48

